# Supplementary material for: Threat reduction must be coupled with targeted recovery programmes to conserve global bird diversity
Source: Nat Ecol Evol. 2025 Jun 24;9(8):1499–511. doi: 10.1038/s41559-025-02746-z (PMC12328217; doi:10.1038/s41559-025-02746-z)
Supplement: Supplementary file 2 — Reporting Summary [file 41559_2025_2746_MOESM2_ESM.pdf]

nature portfolio

Corresponding author(s): Kerry Stewart
Last updated by author(s): May 5, 2025

Reporting Summary

Nature Portfolio wishes to improve the reproducibility of the work that we publish. This form provides structure for consistency and transparency in reporting. For further information on Nature Portfolio policies, see our Editorial Policies and the Editorial Policy Checklist.

Statistics

For all statistical analyses, confirm that the following items are present in the figure legend, table legend, main text, or Methods section.

n/a	Confirmed	
☐	☒	The exact sample size (*n*) for each experimental group/condition, given as a discrete number and unit of measurement
☐	☒	A statement on whether measurements were taken from distinct samples or whether the same sample was measured repeatedly
☐	☒	The statistical test(s) used AND whether they are one- or two-sided<br>*Only common tests should be described solely by name; describe more complex techniques in the Methods section.*
☐	☒	A description of all covariates tested
☐	☒	A description of any assumptions or corrections, such as tests of normality and adjustment for multiple comparisons
☐	☒	A full description of the statistical parameters including central tendency (e.g. means) or other basic estimates (e.g. regression coefficient) AND variation (e.g. standard deviation) or associated estimates of uncertainty (e.g. confidence intervals)
☐	☒	For null hypothesis testing, the test statistic (e.g. F, t, r) with confidence intervals, effect sizes, degrees of freedom and P value noted<br>*Give P values as exact values whenever suitable.*
☐	☒	For Bayesian analysis, information on the choice of priors and Markov chain Monte Carlo settings
☒	☐	For hierarchical and complex designs, identification of the appropriate level for tests and full reporting of outcomes
☐	☒	Estimates of effect sizes (e.g. Cohen's *d*, Pearson's *r*), indicating how they were calculated

Our web collection on statistics for biologists contains articles on many of the points above.

Software and code

Policy information about availability of computer code

Data collection	No software was used to collect data for this study. Data was downloaded from publicly available sources cited in the manuscript and detailed in the data availability statement.
Data analysis	R version 4.2.2 was used to conduct analyses. R packages used in the analyses (with their versions) are cited in the manuscript. Code used to run analyses and produce figures is provided in the FigShare repository: https://doi.org/10.6084/m9.figshare.26067970, as detailed in the Code Availability statement in the manuscript.

For manuscripts utilizing custom algorithms or software that are central to the research but not yet described in published literature, software must be made available to editors and reviewers. We strongly encourage code deposition in a community repository (e.g. GitHub). See the Nature Portfolio guidelines for submitting code & software for further information.

Data

Policy information about availability of data

All manuscripts must include a data availability statement. This statement should provide the following information, where applicable:

- Accession codes, unique identifiers, or web links for publicly available datasets
- A description of any restrictions on data availability
- For clinical datasets or third party data, please ensure that the statement adheres to our policy

AVONET data on morphological, ecological and geographical traits for all birds is available for use under the creative commons licence (CC BY 4.0): https://

doi.org/10.6084/m9.figshare.16586228.v7. Data on IUCN extinction risk categories, and threats affecting each species, are available from the IUCN Red List and can be accessed through the package rredlist. Information on terms of use of IUCN Red List data can be found at https://www.iucnredlist.org/terms/terms-of-use. Code used for figures and analyses and supplementary datasets are presented in https://doi.org/10.6084/m9.figshare.26067970.

Research involving human participants, their data, or biological material

Policy information about studies with human participants or human data. See also policy information about sex, gender (identity/presentation), and sexual orientation and race, ethnicity and racism.

Reporting on sex and gender	n/a
Reporting on race, ethnicity, or other socially relevant groupings	n/a
Population characteristics	n/a
Recruitment	n/a
Ethics oversight	n/a

Note that full information on the approval of the study protocol must also be provided in the manuscript.

Field-specific reporting

Please select the one below that is the best fit for your research. If you are not sure, read the appropriate sections before making your selection.

☐ Life sciences ☐ Behavioural & social sciences ☒ Ecological, evolutionary & environmental sciences

For a reference copy of the document with all sections, see nature.com/documents/nr-reporting-summary-flat.pdf

Ecological, evolutionary & environmental sciences study design

All studies must disclose on these points even when the disclosure is negative.

Study description	Our study uses existing datasets to compare outcomes between biodiversity metrics (functional richness and species richness) under different conservations scenarios for extant birds. Extinction risk was modelled using a phylogenetic generalized linear mixed model using expected population decline from threats (as listed by the IUCN [2022]) as fixed effects, and phylogeny and spatial variables as random effects. The extinction risk model was used to apply a range of extinction scenarios, describing varying degrees of threat reduction. 9873 extant birds were included representing 89% of the extant avian assemblage.
Research sample	The sample-size was determined by the number of species for which there was phylogenetic, spatial and morphological data, accounting for mismatches between taxonomies so as to avoid repeating data between synonyms. Our sample includes 89% of extant birds globally. Phylogenetic data were obtained from Jetz et al. (2012), and morphological data and spatial data were obtained from AVONET (Tobias et al., 2021). Data on threats affecting bird species were obtained from the IUCN Red List (IUCN, 2022).
Sampling strategy	All birds for which we had phylogenetic, spatial and morphological data were included. We accounted for mismatches between taxonomies so as to avoid repeating data between synonyms. This is described in detail in the Methods and Supplementary Information provided with the manuscript.
Data collection	We used publicly available datasets, detailed in the Methods and Data Availability statement of the manuscript.
Timing and spatial scale	Global. Present and project 100 years into the future.
Data exclusions	A matching procedure was used to translate between BirdLife (threat and morphological data, used by Tobias et al. [2021] and IUCN [2022]) and BirdTree (used by Jetz et al., 2012) taxonomies. This was needed to enable analysis of functional diversity loss whilst accounting for phylogenetic covariance between species. Of the 11 003 extant birds listed in the BirdLife taxonomy in 2023, 9873 synonyms were included. The impact of including all synonyms has been assessed and is detailed in the Supplementary Information.
Reproducibility	We have made our code available via a Figshare repository (https://doi.org/10.6084/m9.figshare.26067970). We have stated packages (including their versions) used, and included information on arguments used, and priors used for Markov-Chain Monte Carlo models. In the code we provide seed values where appropriate so that the analyses can be reproduced exactly.
Randomization	This was not an experimental study so no samples, organisms or participants were used.
Blinding	This was not an experimental study so no blinding was used.

Did the study involve field work? ☐ Yes ☒ No

Reporting for specific materials, systems and methods

We require information from authors about some types of materials, experimental systems and methods used in many studies. Here, indicate whether each material, system or method listed is relevant to your study. If you are not sure if a list item applies to your research, read the appropriate section before selecting a response.

Materials & experimental systems

n/a	Involved in the study
☒	☐ Antibodies
☒	☐ Eukaryotic cell lines
☒	☐ Palaeontology and archaeology
☒	☐ Animals and other organisms
☒	☐ Clinical data
☒	☐ Dual use research of concern
☒	☐ Plants

Methods

n/a	Involved in the study
☒	☐ ChIP-seq
☒	☐ Flow cytometry
☒	☐ MRI-based neuroimaging

Plants

Seed stocks	n/a
Novel plant genotypes	n/a
Authentication	n/a
